# Practical Notes on Diagnosis and Treatment

**Published:** 1916-07-08

**Authors:** 


### PRACTICAL NOTES ON DIAGNOSIS AND TREATMENT.

**Blood-Poisoning.**

In modern days there is perhaps some danger of overlooking the value of full doses of perchloride of iron. Besides erysipelas there are many febrile states due to a general infection in which the perchloride is valuable—severe tonsillitis, puerperal fever, septic meningitis, &c.

**Habitual Abortion.**

Perhaps the fact is difficult to explain, but it is beyond question that in women who have had a series of abortions the steady administration of potassium chlorate during a subsequent pregnancy is sometimes followed by the birth of a full-time child. A dose of five grains may be given thrice daily.

**Dilatation of the Stomach.**

If the patient is given 120 grains of sodium bicarbonate dissolved in half a tumblerful of water, and this is followed by 90 grains of tartaric acid, also dissolved in water, the carbonic-acid gas set free will distend the stomach, and the size of the organ can readily be defined by inspection, palpation and percussion.

**Reflexes in Hysteria.**

In hysteria the knee-jerks are often exaggerated; ankle clonus is not common, but it may occur; the plantar reflex is frequently absent; and the abdominal reflexes also may be absent. The one definite response which negatives functional nervous disease is Babinski's sign—hyper-extension of the great toe on stimulating the sole.

**Intermittent Claudication.**

In this condition there is pain in the calves on walking. The patient starts without discomfort, but soon severe pain seizes him, and he is obliged to stop. It is associated with degenerative changes in the tibial arteries, and is very resistant to treatment. Potassium iodide and abstinence from tobacco sometimes appear to be beneficial.

**Pneumothorax.**

This occurring in a patient without history of pulmonary disease must needs give rise to a suspicion of tubercle as the underlying cause. Nevertheless, there are cases in which no further trouble develops. These so-called "idiopathic" cases usually occur during exertion. Under the influence of rest followed after a time by respiratory exercises such patients get perfectly well.

**Tabetic Pains.**

For the relief of these, antipyrin, phenacetin, and acetanilide are all useful. They are most effective where given in full doses, and as soon as ever the attack of pain threatens. Another practical point is that immediately the dose is taken the patient should lie down for a time. Sir William Gowers recommends aluminium chloride in five to ten grain doses thrice daily as tending to lessen the frequency and severity of the pains of tabes.

**Diabetes Insipidus.**

This is a condition very difficult to treat with success. In some cases large doses of antipyrin have done good, in others hypodermic injections of strychnine.

**Angina Pectoris.**

In young adults angina pectoris may depend on syphilitic changes at the root of the aorta or in the coronary arteries, but it sometimes occurs, even in a fatal form, in men under thirty, and without any discoverable cardiac or vascular lesion.

**Infantile Syphilis.**

Mercurial treatment, as inunction or grey powder internally, should be continued for at least six months after the disappearance of symptoms; in cases where there are gummata or signs of epiphysitis, potassium iodide in addition to the mercury should be ordered.

**Pernicious Anæmia.**

The natural variation in the course of this disease makes it difficult to judge with confidence the effects of remedies. It seems, however, fairly certain that in addition to arsenic, bone marrow has some value, even in severe cases. Dr. Byrom Bramwell has recently used salvarsan with some success.

**Enteric Fever.**

Most authorities agree that the steady administration of chlorine water is beneficial in enteric fever. It is prepared by putting 30 grains of potassium chlorate into a 12-ounce bottle, adding 40 minims of strong hydrochloric acid, then pouring in water gradually, and shaking after each addition. An ounce of this with three grains of quinine may be given every three hours, and continued until convalescence is established.

**Movable Kidney.**

In a large proportion of cases of movable kidney the patient is quite unaware of the condition; she suffers from no symptoms which may be attributed to movable kidney, and it is far better to leave her in ignorance, otherwise her imagination may be quickened to the point of hysteria. Perhaps some of the patient's friends should be told lest perchance the discovery of the condition at a later date may suggest the development of something new.

**Diabetic Coma.**

Often when diabetic coma comes on it is found that sugar has disappeared from the urine, and if the comatose patient is not known to have suffered from diabetes there is danger that the true diagnosis will be overlooked. The ferric-chloride test will always, in such cases, show the presence of di-acetic acid. On the other hand, though rarely, a central nervous lesion—say, cerebral hæmorrhage—may cause both coma and glycosuria, the combination obviously suggesting diabetic coma; but here the ferric-chloride test will be negative.

